# Supplementary material for: Enthalpy-entropy compensation of atomic diffusion originates from softening of low frequency phonons
Source: Nat Commun. 2020 Aug 7;11:3977. doi: 10.1038/s41467-020-17812-2 (PMC7414111; doi:10.1038/s41467-020-17812-2)
Supplement: Supplementary file 1 — Supplementary Information [file 41467_2020_17812_MOESM1_ESM.pdf]

Supplementary information for

Enthalpy-entropy compensation of atomic diffusion originates from softening of low frequency phonons

Simon Gelin[1,2,3], Alexandre Champagne-Ruel[1], and Normand Mousseau[1]

[1]Département de physique and Regroupement québécois sur les matériaux de pointe, Université de Montréal, C.P. 6128, Succursale Centre-Ville, Montréal, Québec H3C3J7, Canada
[2]Département de mathématiques et de génie industriel, École Polytechnique de Montréal, Montréal, Québec H3C3A7, Canada
[3]Institut Lumière Matière, UMR5306 Université Lyon 1-CNRS, Université de Lyon, F-69622 Villeurbanne Cedex, France

Supplementary Text

Experimental data in silicon and aluminum

In silicon single crystals, Cu [1], Fe [2, 3], Ni [4], O [5], Li [6, 7, 8, 9] diffuse via the direct interstitial mechanism, C [10] diffuses via the kick-out mechanism, Al [11] and Si-I [12] via the interstitialcy mechanism, and Si-V [12] via the vacancy mechanism. Among species for which we report experimental data in aluminium single crystals, H [13] is the only one diffusing via the direct interstitial mechanism. All other impurities diffuse through the vacancy mechanism (Ti, V, Cr, Mn, Fe, Co, Ni, Cu, Zn, Mg, Si, Ga are taken from Du *et al.* [14]; Al, Sc, Zr, Mo, Hf, W from Knipling *et al.* [15]; Ag, Au, Cd, In, Sn from Le Claire and Neumann [16]; and Ge from Rummel *et al.* [17]).
Diffusion coefficients collected for silicon are displayed in Supplementary Figure S1, and all diffusion parameters in silicon and aluminium are reported in Supplementary Table S1.

Supplementary Figures

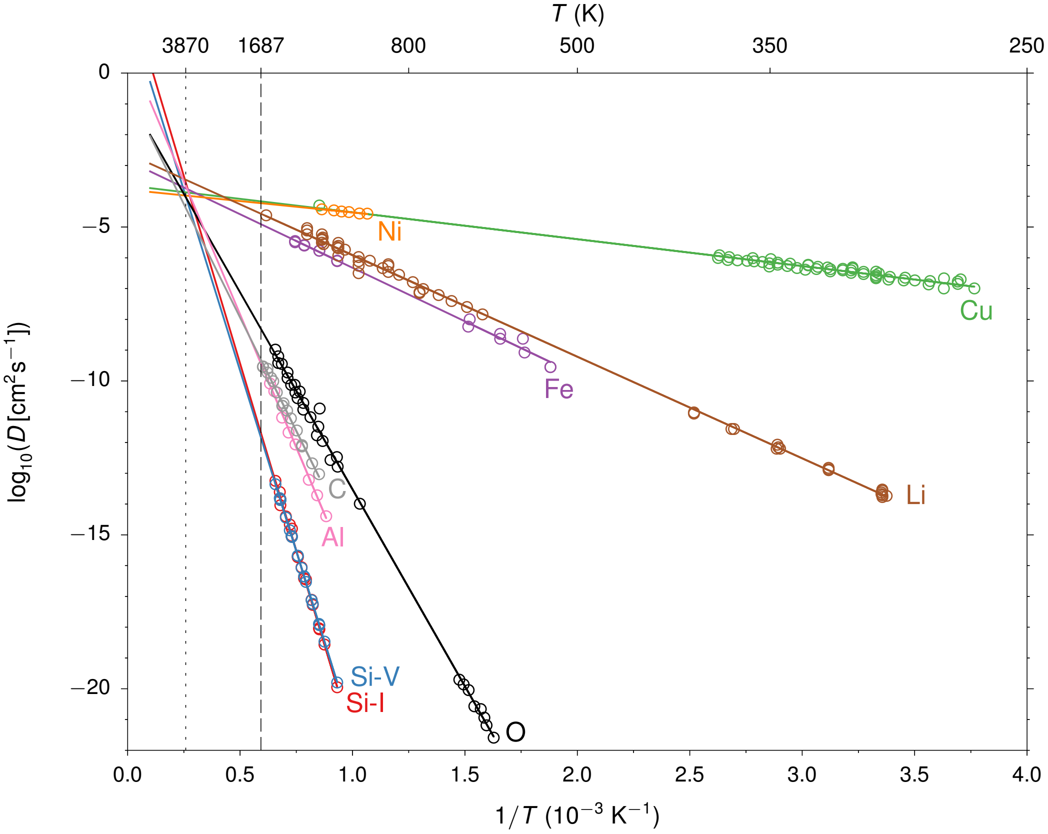

Figure S1: **Arrhenius plot of self- and impurity diffusion coefficients in silicon.** Circles represent diffusion coefficients extracted from the literature (see above Supplementary Information Text section for references). The vertical dashed line marks the melting temperature of silicon at zero pressure, and straight lines are fits obtained using the Arrhenius law. Due to the compensation effect, they all cross at around the temperature indicated by the vertical dotted line, and given by $(k_B\gamma_c)^{-1} \simeq 3870\,\mathrm{K}$ (γ_c(c-Si) $= 3.0\,\mathrm{eV}^{-1}$, see Main Text).

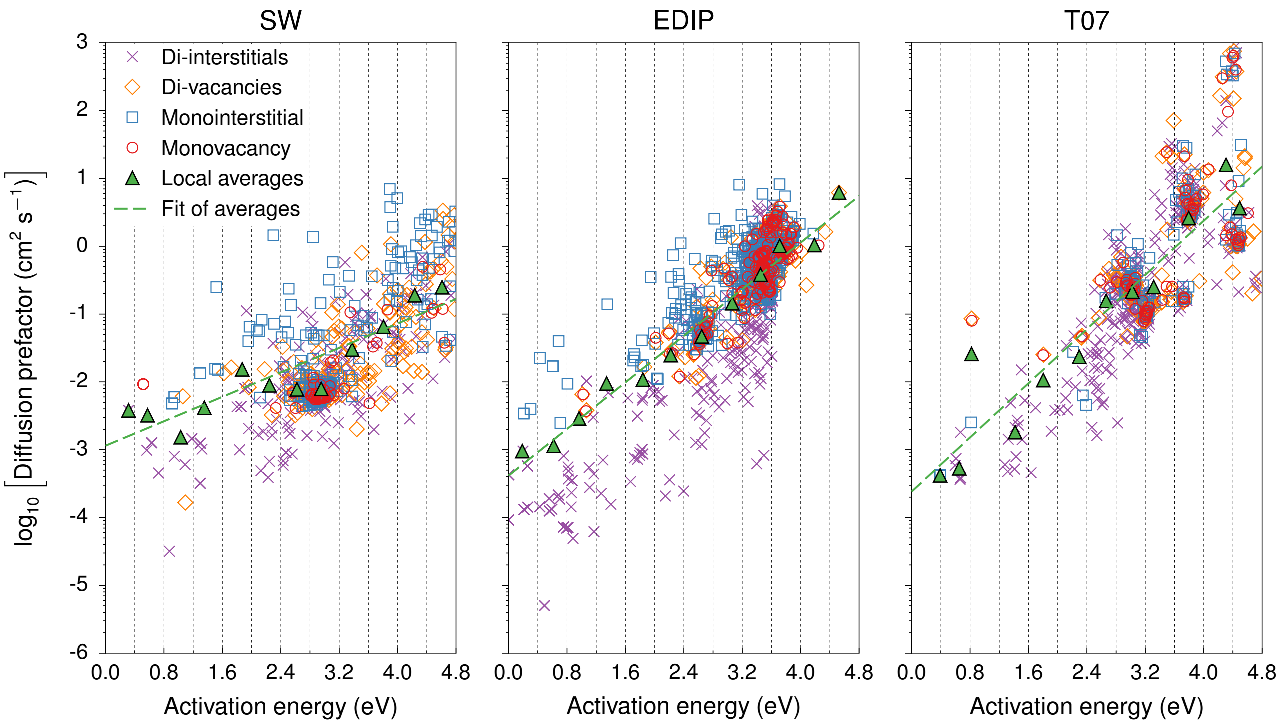

Figure S2: **Raw numerical diffusion data in crystalline silicon.** Each open symbol represents the Arrhenius diffusion parameters, computed as indicated in Main Text, of activated events probed in crystalline silicon configurations modeled with the SW, EDIP, and modified Tersoff (T07) potentials. As indicated in the legend, activated events are sampled in configurations containing vacancy or interstitial defects (the legend applies to all subfigures). Per-event data is averaged over energy bins of width 0.4 eV, from 0 to 4.8 eV. Then, the resulting local averages (filled green triangles) are fitted with the compensation law (green straight dashed line) to extract compensation parameters.

Supplementary Tables

Host	Impurity	D_0 (cm^2 s^{-1})	Activation energy (eV)	Impurity	D_0 (cm^2 s^{-1})	Activation energy (eV)
Si (exp.)	Ni	1.6×10^{-4}	0.15	C	2.7×10^{-1}	2.92
	Cu	2.3×10^{-4}	0.17	Al	6.5	3.43
	Li	2.4×10^{-3}	0.66	Si-V	114	4.65
	Fe	1.4×10^{-3}	0.69	Si-I	547	4.83
	O	1.9×10^{-1}	2.54			
Al (exp.)	H	1.75×10^{-4}	0.168	Ni	4.1	1.50
	Zn	1.19×10^{-1}	1.20	Co	192	1.74
	Au	1.31×10^{-1}	1.21	Sc	5.31	1.79
	Ag	1.18×10^{-1}	1.21	Mn	135	2.19
	Si	1.38×10^{-1}	1.22	Fe	3.620	2.22
	Sn	8.4×10^{-1}	1.23	Hf	107	2.50
	Ge	3.39×10^{-1}	1.24	Zr	728	2.51
	Mg	1.49×10^{-1}	1.25	W	10.6	2.58
	Ga	4.9×10^{-1}	1.27	Mo	14.0	2.59
	In	1.16	1.27	Ti	1.120	2.69
	Cd	1.04	1.29	Cr	6.750	2.71
	Al	1.37×10^{-1}	1.29	V	16.000	3.13
	Cu	4.44×10^{-1}	1.39			
Al (DFT)	H	1.0×10^{-3}	0.14	O	4.92×10^{-1}	1.16
	B	6.64×10^{-2}	0.75	Cu	4.37×10^{-2}	1.25
	N	5.7×10^{-3}	0.91	Mg	1.19×10^{-1}	1.27
	Si	3.66×10^{-2}	1.15	Al	6.6×10^{-2}	1.29

Table S1: **Arrhenius diffusion parameters extracted from the literature.** D_0 is the diffusion pre-exponential factor. All experimental references are given in the Supplementary Text above, and numerical ones in Methods.

	c-Si	a-Si	CuZr	$Ni_{80}P_{20}$	LJ
Preparation of amorphous solids inherent states					
Nb. of atoms, N_{at}	4096	4096	3456	4000	6000
Nb. of samples, N_{s}		50	50	50	50
T_{eq} (K)		1800	1200	800	$\simeq$ 1220
T_{relax} (K)		800	600	400	$\simeq$ 977
Quench rate, q (K ns^{-1})		2000	10	100	$\simeq$ 0.74
Relaxation time, t_{relax} (ns)		100	600	1000	$\simeq$ 3280
Generation and analysis of activated events					
Nb. activation centers, N_{ac}		400	250	250	400
Activation radius, R_{a} (Å)	3.0	3.5	4.5	4.0	7.5
Nb. of events, N_{ev}		143,552	244,794	133,502	102,142
$f^{\mathrm{IS}}_{\mathrm{max}}$ (eV Å^{-1})	3×10^{-8}	3×10^{-8}	5×10^{-5}	5×10^{-5}	1.2×10^{-10}
$f^{\ddagger}_{\mathrm{max}}$ (eV Å^{-1})	1×10^{-7}	1×10^{-7}	5×10^{-5}	5×10^{-5}	1.2×10^{-8}
ΔE_{con} (eV)	1×10^{-6}	1×10^{-6}	1×10^{-4}	1×10^{-4}	3×10^{-7}
Δr_{con} (Å)	1×10^{-4}	1×10^{-4}	1×10^{-1}	1×10^{-1}	2.5×10^{-5}
ΔE_{dup} (eV)	1×10^{-2}	1×10^{-6}	1×10^{-4}	1×10^{-5}	3×10^{-8}
Δr_{dup} (Å)		1×10^{-4}	1×10^{-2}	1×10^{-2}	2.5×10^{-5}
δ (Å)	1×10^{-7}	1×10^{-7}	1×10^{-8}	1×10^{-8}	2.5×10^{-6}

Table S2: **Properties of studied systems and activated events datasets.** Parameters are all defined in Methods, except the number of events, N_{ev}, which is the total number of events over all 50 inherent states for each type of amorphous solid. Those entries that are not relevant for silicon crystalline configurations with defects are left blank.

Supplementary References

[1] Istratov, A. A., Flink, C., Hieslmair, H., Weber, E. R. & Heiser, T. Intrinsic diffusion coefficient of interstitial copper in silicon. *Physical Review Letters* **81**, 1243–1246 (1998).

[2] Isobe, T., Nakashima, H. & Hashimoto, K. Diffusion coefficient of interstitial iron in silicon. *Japanese Journal of Applied Physics* **28**, 1282–1283 (1989).

[3] Schwalbach, P. *et al.* Diffusion and isomer shift of interstitial iron in silicon observed via in-beam Mössbauer spectroscopy. *Physical Review Letters* **64**, 1274–1277 (1990).

[4] Lindroos, J. *et al.* Nickel: A very fast diffuser in silicon. *Journal of Applied Physics* **113**, 204906 (2013).

[5] Mikkelsen, J. C. The diffusivity and solubility of oxygen in silicon. *MRS Proceedings* **59**, 19–30 (1985).

[6] Fuller, C. S. & Ditzenberger, J. A. Diffusion of lithium into germanium and silicon. *Physical Review* **91**, 193–193 (1953).

[7] Severiens, J. C. & Fuller, C. S. Mobility of impurity ions in germanium and silicon. *Physical Review* **92**, 1322–1323 (1953).

[8] Pell, E. M. Diffusion rate of Li in Si at low temperatures. *Physical Review* **119**, 1222–1225 (1960).

[9] Pell, E. M. Diffusion of Li in Si at high T the isotope effect. *Physical Review* **119**, 1014–1021 (1960).

[10] Rollert, F., Stolwijk, N. & Mehrer, H. Diffusion of carbon-14 in silicon. *Materials Science Forum* **38-41**, 753–758 (1991).

[11] Krause, O., Ryssel, H. & Pichler, P. Determination of aluminum diffusion parameters in silicon. *Journal of Applied Physics* **91**, 5645–5649 (2002).

[12] Südkamp, T. & Bracht, H. Self-diffusion in crystalline silicon: A single diffusion activation enthalpy down to 755 °C. *Physical Review B* **94**, 125208 (2016).

[13] Young, G. A. & Scully, J. R. The diffusion and trapping of hydrogen in high purity aluminum. *Acta Materialia* **46**, 6337–6349 (1998).

[14] Du, Y. *et al.* Diffusion coefficients of some solutes in fcc and liquid Al: critical evaluation and correlation. *Materials Science and Engineering: A* **363**, 140–151 (2003).

[15] Knipling, K. E., Dunand, D. C. & Seidman, D. N. Criteria for developing castable, creep-resistant aluminum-based alloys – a review. *Zeitschrift für Metallkunde* **97**, 246–265 (2006).

[16] LeClaire, A. D. & Neumann, G. 3.2.13 aluminum group metals. In *Landolt-Börnstein - Group III Condensed Matter*, 151–156 (Springer-Verlag, 151–156 1990).

[17] Thürer, A., Rummel, G., Zumkley, T., Freitag, K. & Mehrer, H. Temperature and pressure dependence of Ge diffusion in aluminium. *Physica Status Solidi (a)* **149**, 535–547 (1995).
